# Sex‐dependent effects of environmental enrichment on mood‐related behaviors, central and peripheral BDNF, and adrenal catecholamine enzymes in mice

**DOI:** 10.14814/phy2.70870

**Published:** 2026-04-14

**Authors:** M. F. Herselman, S. Y. Luo, L. Y. Lin, X. F. Zhou, L. Bobrovskaya

**Affiliations:** ^1^ School of Pharmacy and Biomedical Science, College of Health Adelaide University Adelaide South Australia Australia; ^2^ Department of Breast Surgery Xiangya Hospital Changsha Hunan China; ^3^ Health and Biomedical Innovation, School of Pharmacy and Biomedical Science, College of Health Adelaide University Adelaide South Australia Australia; ^4^ Center for Pharmaceutical Innovation, School of Pharmacy and Biomedical Science, College of Health Adelaide University Adelaide South Australia Australia

**Keywords:** anxiety, BDNF, catecholamines, environmental enrichment, sex differences

## Abstract

Housing conditions, particularly environmental enrichment (EE), can influence experimental outcomes and welfare. While EE is generally regarded as beneficial, a male bias exists in research supporting this. This study investigated whether sex differences exist in levels of BDNF in the brain and peripheral tissues in environmentally enriched mice. Expression of the catecholamine biosynthetic enzymes of the adrenal glands, key to the sympathoadrenal medullary system and stress response, was also investigated. We showed that female mice exposed to EE exhibited increased anxiety‐like behaviors. EE in male mice did not induce anxiety‐like behavior, and it was associated with increased hippocampal and pituitary BDNF expression, suggestive of enhanced neurotrophic support. In the adrenal gland, the levels of adrenal catecholamine biosynthetic enzymes, specifically total tyrosine hydroxylase and PNMT levels, were increased in females, but not in males. In conclusion, EE may serve as a mild stressor in female mice. In male mice, EE may have induced neurotrophic support of the hippocampus since hippocampal BDNF levels were increased with minimal changes to adrenal catecholamine synthetic enzymes. This study highlights the importance of considering sex as a biological variable in translational neuroscientific research.

## 1 INTRODUCTION

The impact of housing conditions on animal models in research is often an overlooked factor when designing experiments. In recent years, it has become evident that housing conditions can have both physiological and psychological impacts on experimental animals (Cait et al., 2022). Typical housing conditions of experimental rodents, for example, include group housing in a small cage with ad libitum access to food and water as well as sufficient bedding material; however, these conditions restrict natural behaviors of climbing, burrowing, and exploration (Cait et al., 2022; Ratuski & Weary, 2022). Thus, researchers have developed modifications to animal husbandry such as environmental enrichment (EE) which aims to improve physiological and psychological wellbeing by providing the research animals access to stimuli such as natural materials, tunnels, ropes, running wheels, ramps, and other toys (Cait et al., 2022; Lopes et al., 2017). Many studies of neurodegenerative disease include EE as an intervention due to its proven ability to increase neurogenesis, which is particularly relevant in conditions such as anxiety and depression where neurogenesis is negatively affected (Macartney et al., 2022). However, a recent systematic review of the effect of EE on the welfare of laboratory rodents found strong bias in the sex of rodents, with most studies in mice only including male mice (Mieske et al., 2022). This causes concern for the translatability of neuroscience research incorporating EE since, in humans, disorders such as anxiety and depression show a higher incidence in females (Javaid et al., 2023; Shi et al., 2021; Shorey et al., 2022).

While EE is primarily used to improve welfare, many studies validate its efficacy through the assessment of anxiety‐like behaviors in rodents. EE was recently shown to increase anxiety‐like behavior in male Balb/C mice, a strain with high sensitivity to altered environmental stimuli (de Brito et al., 2025). Other studies have shown similar increases in anxiety‐like behavior in male C57BL6J mice (dos Anjos‐Garcia et al., 2022), as well as anxiolytic effects in male mice of various strains (Benaroya‐Milshtein et al., 2004; Rogers et al., 2017). Studies that evaluated anxiety‐like behaviors in both sexes report variable outcomes too. Hendershott et al. (2016) showed that female C57BL6J mice had a greater preference for the open arms in the elevated plus‐maze, regardless of housing conditions, compared to males. Conversely, another study showed anxiogenic effects of EE in female C57BL6J mice, but an anxiolytic effect in EE males (Lin et al., 2011), and another showed anxiolytic effects in both male and female Sprague–Dawley rats (Peña et al., 2006). Clearly, behavioral outcomes of EE are highly variable between studies, making interpretation challenging given some studies reported behavior without accompanying molecular data (Hendershott et al., 2016; Peña et al., 2006).

Nonetheless, EE has been shown to positively influence neurotrophin levels in the brain, such as brain‐derived neurotrophic factor (BDNF) implicated in synaptic plasticity (Costa et al., 2023; Ismail et al., 2024; Keifer, 2022). BDNF is decreased in the brain in neurodegenerative diseases as well as in conditions such as anxiety and depression (Rana et al., 2021; Zhu et al., 2019). proBDNF, the precursor neurotrophin that is cleaved by matrix metalloproteases into BDNF, has been reported by our laboratory and others in higher levels in models of anxiety and depression (Li et al., 2023; Lin et al., 2020; Zhong et al., 2019). Studies reporting on the effects of EE on proBDNF levels in the brain provide inconsistent findings (Giacobbo et al., 2019; Novkovic et al., 2015; Vazquez‐Sanroman et al., 2013), which may be related to different EE protocols, but may also be due to differential modulation of proBDNF levels in different brain regions. Interestingly, EE was previously shown to enhance the conversion of proBDNF to BDNF in the adult rat hippocampus, which may be related to increased physical activity (Cao et al., 2014), and highlights how the choice of enrichment items can influence the brain. Studies have reported increased hippocampal BDNF levels in adult male mice and rats; however, female hippocampal BDNF levels were unaffected, suggesting sex‐dependent effects and warranting further study of the mechanisms involved (Marion et al., 2022; Vinogradova et al., 2023). The age of experimental animals may also play a role in the effects of environmental enrichment since Sakhaie et al. (2020) showed that EE adolescent mice had increased hippocampal BDNF levels in both sexes.

EE has also been reported to modulate BDNF and proBDNF levels in the hypothalamus (Aldhshan & Mizuno, 2022), a region where BDNF signaling may be important for regulating the stress response and HPA axis activity. BDNF has been shown to induce corticotropin‐releasing hormone in the hypothalamus (Jeanneteau et al., 2012), while EE has been found to attenuate markers of HPA axis output such as corticosterone in male rodents (Dandi et al., 2023). Furthermore, hypothalamic BDNF expression may exhibit sex‐dependent patterns, with higher levels previously reported in enriched female rats compared to males (Bakos et al., 2009). Given these roles, BDNF may serve as a key mediator of sex‐specific environmental impacts on hypothalamic‐driven pathways.

While not as well studied as the endocrine and behavioral responses to EE, physiological responses to EE are important to characterize. Cardiovascular function is influenced by the autonomic nervous system and the release of catecholamines by the sympathoadrenal medullary system, which stimulates the adrenal medulla to release catecholamines such as adrenaline in response to stress thus increasing blood pressure, heart rate, and hepatic glucose production (Domínguez‐Oliva et al., 2025; Herman et al., 2016). Catecholamine biosynthesis is rate limited in the adrenal medulla by tyrosine hydroxylase (TH), which forms part of a biosynthetic pathway involving a series of enzymes in which TH first converts tyrosine to ʟ‐DOPA, after which DOPA decarboxylase generates dopamine from ʟ‐DOPA, dopamine β–hydroxylase then converts dopamine into noradrenaline, and phenylethanolamine N‐methyltransferase (PNMT) which finally converts noradrenaline to adrenaline (Amano et al., 2013). TH activity is known to be regulated by most forms of regulation, including phosphorylation, and such events occur at serine residues (Ser) 19, 31, 40 (Dunkley & Dickson, 2019). Ser40 (and/or Ser31) are known to be directly involved in the activation of TH (Dunkley & Dickson, 2019). Previous studies have reported inconsistent findings on the effects of EE on TH activity in the adrenal medulla (Marashi et al., 2003, 2004; Van Loo et al., 2002). While literature on adrenal TH activity during EE is lacking, it has been established that stress or anxiety can lead to increased TH activity (Ong et al., 2011). A previous study by Marashi et al. (2003) attributed changes in TH activity in their study to increased aggression in male mice using a long‐term EE protocol, which may be anxiogenic. Indeed, Van Loo et al. (2002) showed that CBA mice developed anxiety following repeated aggression experiences. Clearly, animal strain, age, and sex may contribute to the effects of EE on behavior and neuroendocrine systems. While the roles of BDNF and neuroendocrine systems on physiological homeostasis are well explored, to our knowledge no studies have simultaneously assessed neurotrophic signaling with peripheral markers of the sympathoadrenal medullary system across both sexes. It is therefore difficult to determine whether sex‐specific behavioral responses to EE are influenced by central neurotrophin expression or through modulation of adrenal biosynthetic pathways.

In the present study, we aimed to investigate whether EE has any sex‐dependent effects on behavior and neurotrophin expression in the brain and periphery. We also investigated whether EE and biological sex could affect the catecholamine synthetic enzymes of the adrenal glands with implications for the sympathoadrenal medullary system in C57BL6J mice.

We hypothesized EE would differentially affect behavior and neurotrophin expression in a sex‐dependent manner, and that these effects would be reflected in the expression of adrenal catecholamine biosynthetic enzymes.

## 2 MATERIALS AND METHODS

### 2.1 Animals

Thirty‐two C57BL6 mice pups (Males = 16, Females = 16) were bred in the Reid Building Animal Facility (University of South Australia), maintained under standard conditions (12:12‐h light/dark cycle, lights on between 6 a.m. to 6 p.m., temperature of 22°C ± 1°C, humidity of 52% ± 2%) and housed in groups of 4 per cage with a floor area of 535cm^2^ (Smarttop Emerald Line Individually Ventilated Cages, Techniplast Australia) with standard ad libitum access to water and standard rat and mouse chow (catalogue # SF00‐100, Specialty Feeds, Australia). Cages were selected from a larger cohort of litter‐matched groups to ensure baseline characteristics were balanced across experimental conditions. Males and females were housed separately prior to commencement of the experiment. All mice were acclimatized under identical environmental conditions 2 weeks prior to conducting experiments to minimize cage‐specific effects and were provided with free access to conventional food and water. During this period, the handling of mice was kept to a minimum with only weekly cage cleaning carried out by technical staff as necessary. Following acclimatization, existing cage units of mice were assigned randomly to specific experimental groups and were weighed prior to the commencement of experiments and weekly thereafter. On completion of the experiment and following behavioral testing (described below), all mice were anesthetized and humanely killed with a lethal dose of sodium pentobarbital from 09:00 am at approximately 8 weeks of age. Brain regions including the prefrontal cortex, hippocampus, hypothalamus, and pituitary were then collected similarly to the methods described by Spijker (2011) and (Aboghazleh et al., 2024) along with the adrenal glands and kept at −80°C for further analyses. All animal procedures were in compliance with the protocols approved by the Animal Ethics Committee of the University of South Australia (Ethics number U23‐15). All experimental procedures abide by the Australian Code of Practice for the Care and Use of Animals for Scientific Purposes (National Health and Medical Research Council, 2013), the 3R principles (Tannenbaum & Bennett, 2015), and the ARRIVE guidelines (Percie du Sert et al., 2020).

### 2.2 Environmental enrichment

Thirty‐two 21‐day old C57BL6 mice pups (Males = 16, Females = 16) were allocated into two experimental groups, controls and EE (Male Control = 8, Female Control = 8, Male EE = 8, Female EE = 8) following weaning and the acclimatization period. Pups allocated to the control groups were transferred to new standard housing boxes (535 cm^2^), while the EE groups were transferred to large housing boxes with a floor area of 1050 cm^2^ (ER1050 Emerald Line Individually Ventilated Cages, Techniplast Australia) supplemented with plastic and wooden toys, ladders, tubes, paper boxes, rings, small houses, and a running wheel. Both control groups and EE groups had once‐weekly cage changes and, apart from once‐weekly weighing, handling of all mice was kept to a minimum. Additionally, the environmental enrichment items were rearranged once weekly to prevent habituation. The total protocol for both groups/sexes continued for 4 weeks.

### 2.3 Behavioral testing

Following 4 weeks of the EE protocol, spontaneous locomotor activity and anxiety‐like behavior were assessed using the open field test (OFT) and elevated plus maze test (EPM) when mice were approximately 8 weeks old. All behavioral testing was carried out at 9:00 am under standard overhead room lighting conditions of ~320 lux. The OFT was conducted in a hedged open field of white Plexiglass (40 × 40 × 40 cm). The open field was divided into 5 × 5 cm squares of equal size and the central zone was defined as the central nine of these squares (3 × 3). Each mouse was placed individually in the central zone and allowed to move at will in the open field for a period of 5 min. Entries into each respective zone were defined as the centre of mass of the mouse entering the predefined zone. An overhead digital camera recorded the activity of the mouse during this period and ANY‐maze software was used to analyze the results.

For the EPM test, a cross‐shaped gray Plexiglass apparatus consisting of two closed arms (50 cm in length × 10 cm in width × 40 cm in height) and two open arms (50 cm in length × 10 cm in width) and a central platform (7 cm in length × 7 cm in width) was elevated 50 cm from the floor. Each mouse was placed individually in the centre of the elevated maze oriented with the nose pointing towards the closed arm. An overhead digital camera recorded the number of entries into the different arms (defined as the centre of mass of the mouse entering each arm), the travel distance and the time spent in each arm by each mouse for a period of 5 min and the results were analyzed using ANY‐maze software. All behavioral testing was conducted in a specialized behavioral testing room in the Reid Building Animal Facility (University of South Australia).

### 2.4 Tissue sample preparation

Using the Precellys 24 Tissue Homogenizer (Bertin Technologies, FRA), all tissue samples were subsequently homogenized in ice‐cold RIPA buffer (50 mM Tris, 150 mM NaCl, 1 mM EDTA, 0.5% Triton X‐100, 0.5% Sodium deoxycholate, pH 7.4) containing a protease inhibitor cocktail (catalogue # P8340, Sigma St. Louis, USA). The total protein concentration of the supernatants was assessed by microBCA commercial kit (catalogue # 23235, Thermo Fisher Scientific, USA).

### 2.5 Enzyme‐linked immunosorbent assay

Our laboratory has previously developed a highly specific ELISA assay for the measurement of mBDNF and proBDNF (Lim et al., 2015). The levels of mBDNF and proBDNF were measured in the hippocampus, PFC, hypothalamus, pituitary, and adrenal glands according to the protocols used in the publication by Lim et al. (2015).

### 2.6 Western blot analysis

**FIGURE 1:**
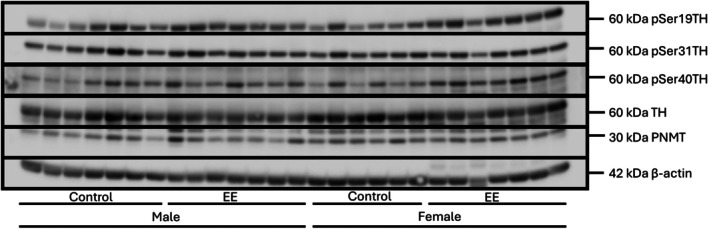
Representative immunoblots for pSer19TH, pSer31TH, pSer40TH, Total TH, PNMT, and β‐Actin. TH, tyrosine hydroxylase; pSer19TH, pSer31TH, pSer40TH: Phosphorylation residues 19, 31, and 40 of tyrosine hydroxylase; PNMT, phenylethanolamine N‐methyltransferase. Complete immunoblots are available in Supplementary Data.

Western blot analysis was performed as previously described (Senthilkumaran et al., 2016). 15 μg of adrenal protein was separated by SDS polyacrylamide gel electrophoresis and subsequently transferred to nitrocellulose membranes (catalogue # 10600016, GE Healthcare, United Kingdom). These were then incubated for 1–1.5 h with 5% skim milk in Tris‐buffered saline containing 0.075% Tween‐20 (TBST), followed by overnight incubation with primary total TH (catalogue # T1299, Sigma‐Aldrich, USA), pSer19TH, pSer40TH, and pSer31TH (homemade ‐produced and validated for specificity as described previously (Gordon et al., 2009)), and PNMT (catalogue # Ab69579, Abcam, Australia) at 4^°^C. These blots were then incubated with the appropriate secondary antibodies (anti‐sheep (catalogue # 713‐035‐147, Jackson ImmunoResearch Laboratories, USA) for pSer40TH, anti‐rabbit (catalogue # 711‐035‐152, Jackson ImmunoResearch Laboratories, USA) for pSer19TH, pSer31TH & PNMT, and anti‐mouse (catalogue # 715‐035‐150, Jackson ImmunoResearch Laboratories, USA) for total TH) for 1 h at room temperature. This was followed by development using a home‐made enhanced chemiluminescence (ECL) detection reagent (10 mL of 100 mM Tris HCL (pH 8.5), 22 μL of 90 mM coumaric acid, 50 μL of 250 mM luminol and 3 μL of H_2_O_2_) which was visualized and quantified using the ImageQuant LAS 4100 imaging system (GE Healthcare, United Kingdom; Figure 1). Densities of the bands were quantified using Image Quant TL software (GE Healthcare Life Sciences, UK). Variability in the loading of protein during gel electrophoresis was corrected by using β‐actin (catalogue # Ab66338, Abcam, Australia) protein levels as a loading control. Site‐specific TH phosphorylation at Ser19, Ser31 and Ser40 were corrected per total TH protein.

### 2.7 Statistical analyses

Sample sizes were determined based on historical data and effect size estimations. Previous studies from our laboratory using the same experimental protocols for Western blotting and ELISA demonstrated that a group size of *n* = 7 is sufficient to identify significant changes in adrenal and neurotrophic markers while minimizing animal usage (Herselman et al., 2023). Additionally, a power analysis (G*Power 3.1) indicated that a total sample size of *n* = 32 (*n* = 8 per group) provides 80% power to detect a large effect size (*f* = 0.52) at *α* = 0.05. For all analyses, the Shapiro–Wilk normality test was used to determine the distribution of the data. Homogeneity of variance was assessed using the Brown‐Forsythe test, with *p* < 0.05 indicating heteroscedasticity. Based on the test results, all variables, except body weight, were confirmed to be non‐normally distributed and heteroscedasticity was present in several outcome variables. Therefore, the use of non‐parametric tests such as the Sheirer‐Ray‐Hare test to evaluate the main effects of sex and environment was justified as a robust uniformly employed analytical approach. This was performed by ranking data across experimental groups and conducting a two‐way ANOVA on the ranks. Sidak's multiple comparisons was used for post‐hoc analysis. All statistical analyses were performed using GraphPad PRISM v7.03 (GraphPad Software, Inc., USA). Since the Scheirer‐Ray‐Hare test is inherently robust to non‐normal distributions and extreme values, formal outlier detection tests were not applied and no data points were excluded from analyses. All western blot data are presented as median with interquartile range of the data expressed as fold change relative to the respective control group and all other data are presented median with interquartile range to facilitate interpretation. Statistical significance was set at *p* < 0.05.

## 3 RESULTS

### 3.1 Body weight

**FIGURE 2:**
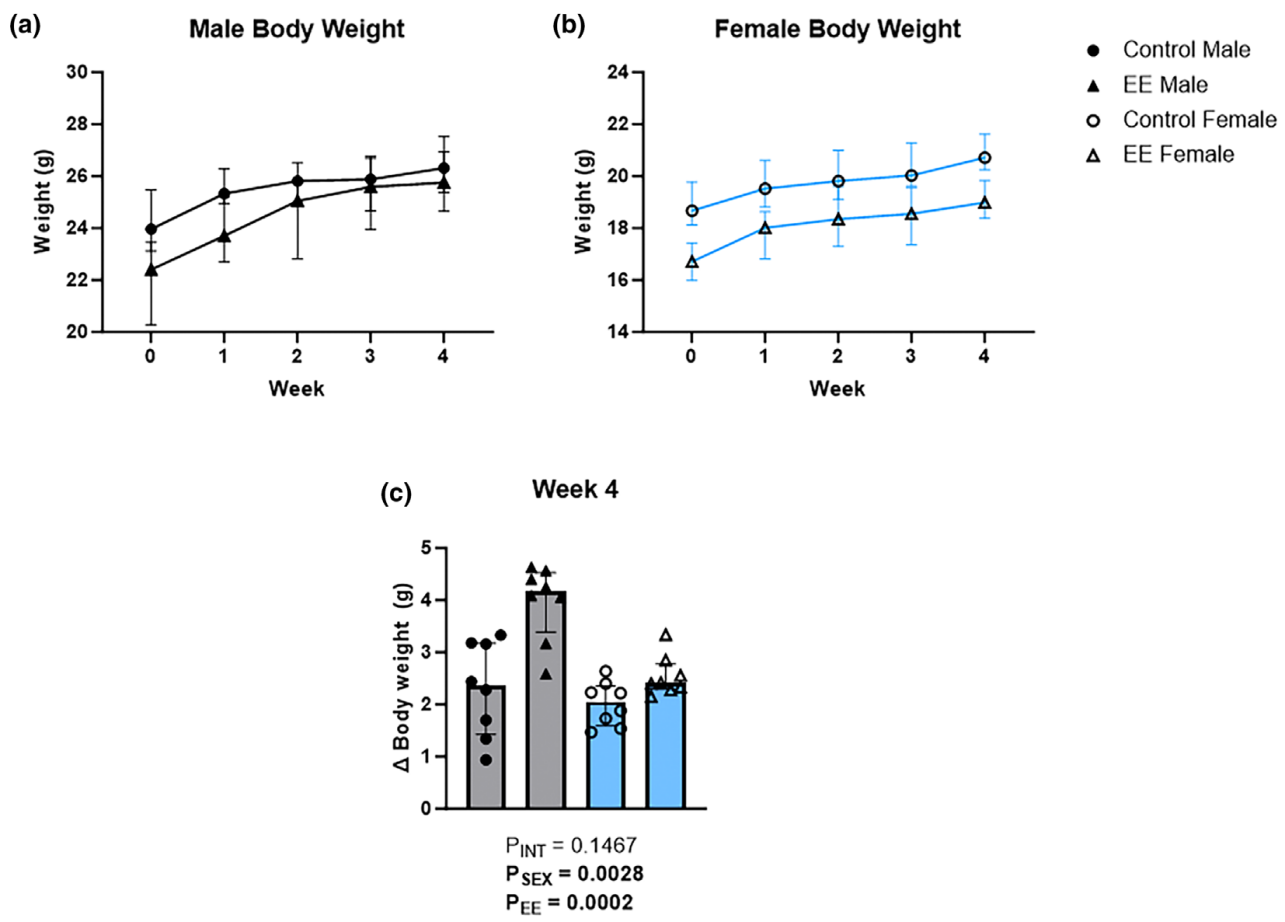
The effects of environmental enrichment (EE) on total body weight in male and female mice. Time course body weight data are presented for each sex (a, b) as well as week 4 delta body weight data (c) (Sheirer‐Ray‐Hare test with Sidak's multiple comparisons). Data are presented as median (IQR). For both males (gray) and females (blue): Control *n* = 8; EE *n* = 8.

Body weight progression throughout the 4‐week protocol is presented in Figure 2a and Figure 2b, with week 4 delta body weight values compared to baseline presented in Figure 2c. At week 4, Sheirer‐Ray‐Hare analysis showed a significant main effect of environment (Figure 2c; H(1) = 9.79, *p* = 0.0002) on delta body weight, as well as a main effect of sex (H(1) = 5.55, *p* = 0.0028) and no significant interaction effect (Figure 2c; H(1) = 1.15, *p* = 0.1467).

### 3.2 Behavior

**FIGURE 3:**
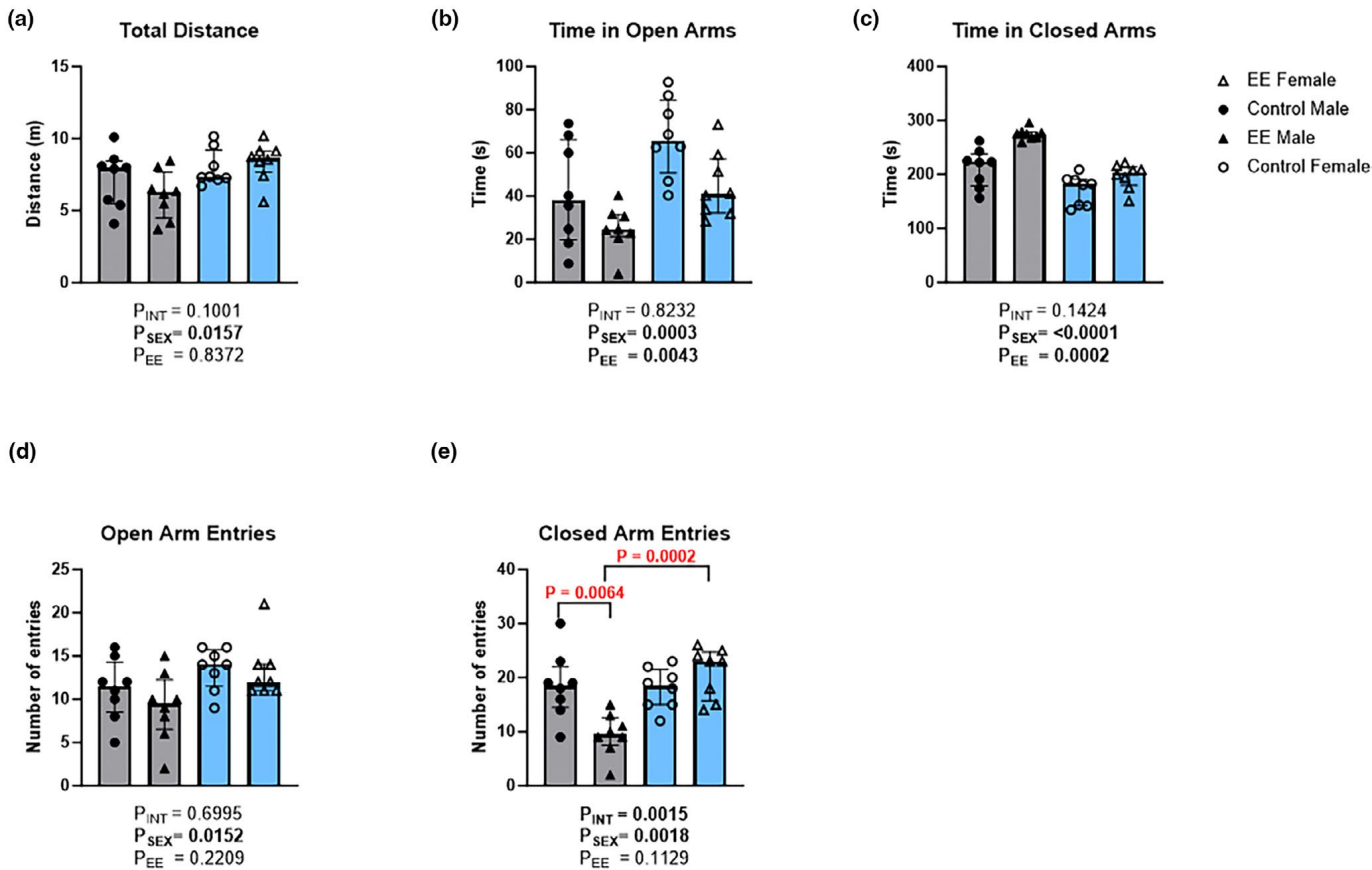
The effects of environmental enrichment (EE) in the EPM test in male and female mice. Measures for total distance (a), time in open arms (b), time in closed arms (c), open arm entries (d), and closed arm entries (e) are shown. Scheirer‐Ray‐Hare test with Sidak's multiple comparisons. Data are presented as median (IQR). For both males (gray) and females (blue): Control *n* = 8; EE *n* = 8.

Anxiety‐like behaviors were assessed using the open field test (OFT) and elevated plus‐maze (EPM). In the EPM, the total distance traveled showed a significant main effect of sex (Figure 3a; H(1) = 5.46, *p* = 0.016), but not environment (H(1) = 0.036, *p* = 0.837) nor an interaction effect (H(1) = 2.39, *p* = 0.100); however, post‐hoc comparisons showed no significant differences in males (*p* = 0.714) or females (*p* = 0.882) exposed to EE, suggestive of no locomotor differences between groups. For the time spent in the open arms, a main effect of sex (Figure 3b; H(1) = 9.67, *p* = 0.0003) and environment (H(1) = 5.46, *p* = 0.004) was found, but no significant interaction effect (H(1) = 0.03, *p* = 0.823). Post‐hoc comparisons showed no significant differences in males (*p* = 0.270) or females (*p* = 0.144) exposed to EE in time spent in the open arms. The measure of time spent in the closed arms of the EPM showed a significant main effect of sex (Figure 3c; H(1) = 13.36, *p* < 0.0001) and environment (H(1) = 6.76, *p* = 0.0002), with no significant interaction effect (H(1) = 0.82, *p* = 0.1424). For the number of entries into the open arms, there was a main effect of sex (H(1) = 5.70, *p* = 0.0152), but not of environment (H(1) = 1.34, *p* = 0.2209) nor an interaction effect (H(1) = 0.13, *p* = 0.6995). A significant main effect of sex (Figure 3e; H(1) = 6.71, *p* = 0.0018) was also evident in the number of closed arm entries in the EPM, as well as an interaction effect (H(1) = 7.00, *p* = 0.0015); however, no significant effect of treatment was found (H(1) = 1.51, *p* = 0.1129). Post‐hoc comparisons revealed a reduction in the number of closed arm entries in male EE mice (median = 9.5; IQR = 12.5–7.5; *p* = 0.0064) compared to male controls (median = 18.5; IQR = 22.0–14.5). The number of closed arm entries was also significantly lower in male EE mice (Median = 9.5; IQR = 12.5–7.5; *p* = 0.0002) compared to female EE mice (median = 23; IQR = 24.75–15.75), despite no differences in the number of entries between male and female controls.

**FIGURE 4:**
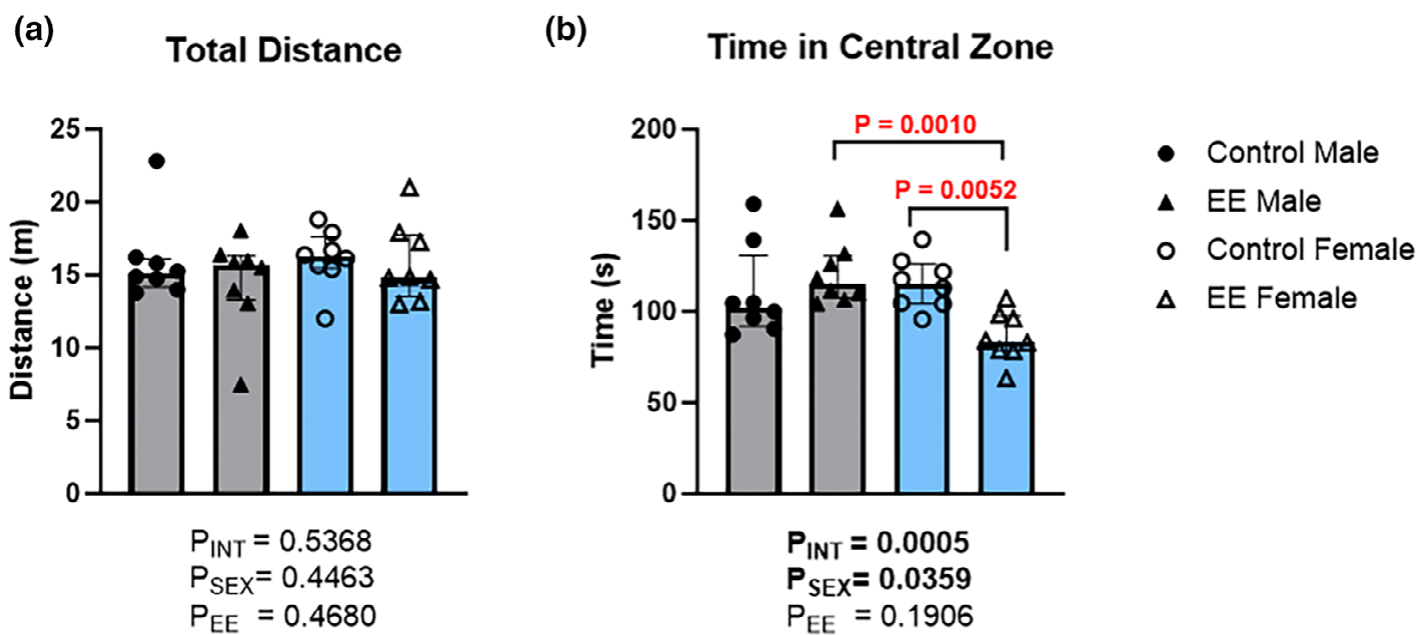
The effects of environmental enrichment (EE) in the open field test (OFT) in male and female mice. Data for measures of total distance (a) and time spent in central zone are shown (b). Scheirer‐Ray‐Hare test with Sidak's multiple comparisons. Data are presented as median (IQR). For both males (gray) and females (blue): Control *n* = 8; EE *n* = 8.

In the OFT, there were no significant main effects for the measure of total distance traveled (Figure 4a; Sex: H(1) = 0.63, *p* = 0.4463; Environment: H(1) = 0.57, *p* = 0.4680; Interaction: H(1) = 0.41, *p* = 0.5368), suggestive of no effects on locomotive behavior. For the time spent in the central zone, a significant main effect of sex (Figure 4b; H(1) = 3.01, *p* = 0.0359), but not environment (H(1) = 1.11, *p* = 0.1906) was found. However, there was also an interaction effect (H(1) = 9.55, *p* = 0.0005), with post‐hoc comparisons showing a significant reduction in the time spent in the central zone in female EE mice (median = 83.50; IQR = 97.65–78.53; *p* = 0.0052) compared to female controls (median = 115.40; IQR = 126.30–104.40). Furthermore, female EE mice spent less time in the central zone (*p* = 0.0010) compared to male EE mice (median = 115.10; IQR = 130.50–107.40).

### 3.3 BDNF and proBDNF

**FIGURE 5:**
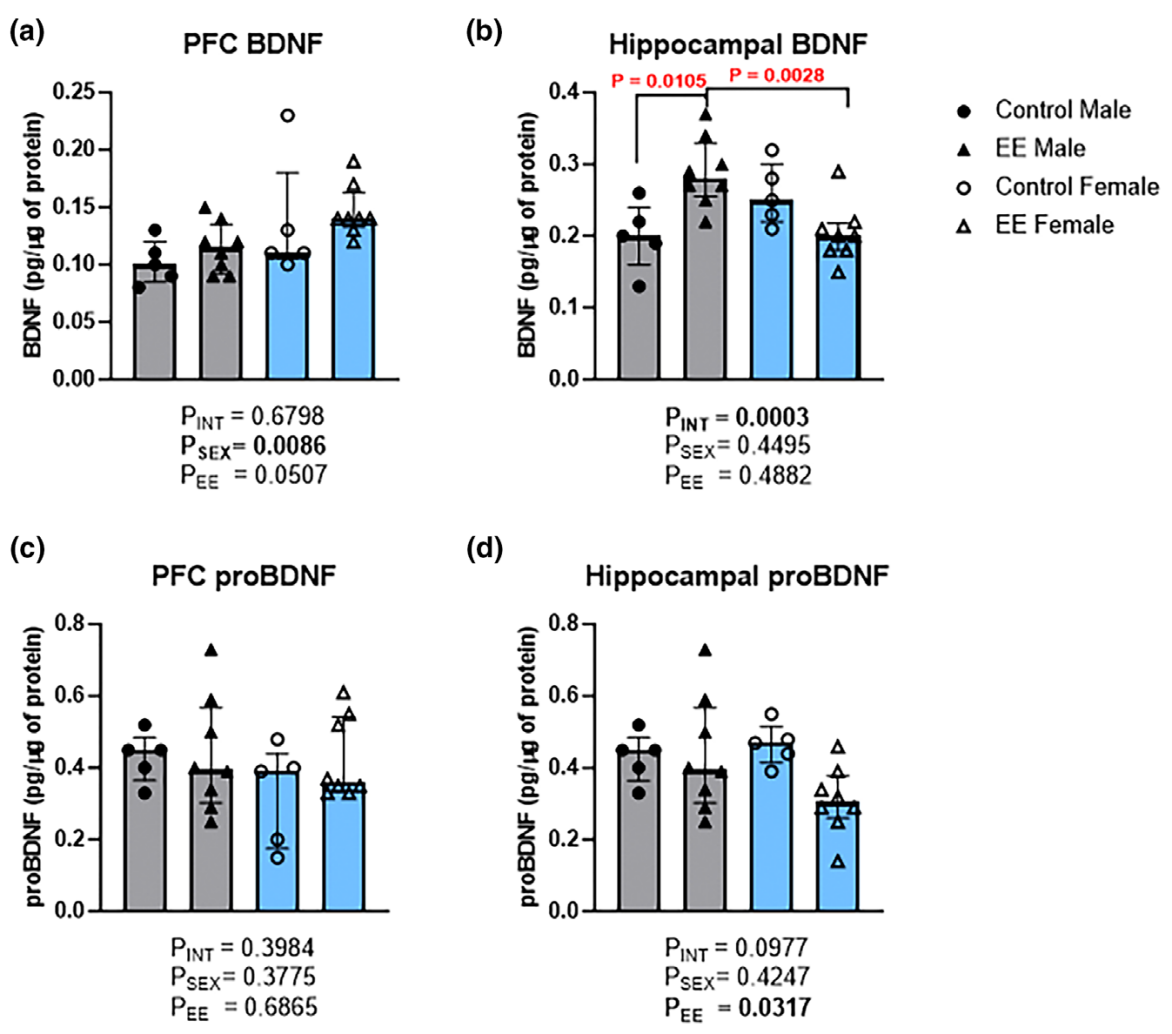
The effects of environmental enrichment (EE) on the expression levels of BDNF and proBDNF in the PFC and hippocampus of male and female mice. Data for PFC BDNF (a), hippocampal BDNF (b), PFC proBDNF (c) and hippocampal proBDNF (d) are shown. Sheirer‐Ray‐Hare test with Sidak's multiple comparisons. Data are presented as median (IQR). Males are shown in gray and females in blue. For all analytes and for both sexes: Control *n* = 5; EE *n* = 8. PFC, prefrontal cortex; brain‐derived neurotrophic factor, BDNF. Small differences in sample sizes were due to inadequate sample volumes and technical issues with tissue collection.

BDNF and proBDNF levels were measured in the PFC and hippocampus of female and male mice. Analysis of BDNF in the PFC showed a significant main effect of sex (Figure 5a; H(1) = 5.98, *p* = 0.0086), with a near‐significant effect of environment (H(1) = 3.07, *p* = 0.0507); however, no significant interaction effect was found (H(1) = 0.13, *p* = 0.6798). Hippocampal BDNF showed no significant main effects of sex (Figure 5b; H(1) = 0.36, *p* = 0.4495) or environment (H(1) = 0.30, *p* = 0.4882); however, there was a significant interaction effect (H(1) = 11.20, *p* = 0.0003). Multiple comparisons showed a significant increase in hippocampal BDNF levels in male EE mice (median = 0.28; IQR = 0.33–0.255; *p* = 0.0105) compared to male controls (median = 0.20; IQR = 0.24–0.16). Male EE mice also had significantly higher BDNF levels (*p* = 0.0028) than female EE mice (Median = 0.20; IQR = 0.22–0.18).

Analysis of proBDNF in the PFC showed no significant main effects or interaction (Figure 5c; Sex: H(1) = 0.86, *p* = 0.3775; Environment: H(1) = 0.18, *p* = 0.6865; Interaction: H(1) = 0.78, *p* = 0.3984). While a significant main effect of environment (Figure 5d; H(1) = 4.26, *p* = 0.0317), but not sex (H(1) = 0.54, *p* = 0.4247), or an interaction effect (H(1) = 2.42, *p* = 0.0977), was evident for hippocampal proBDNF.

**FIGURE 6:**
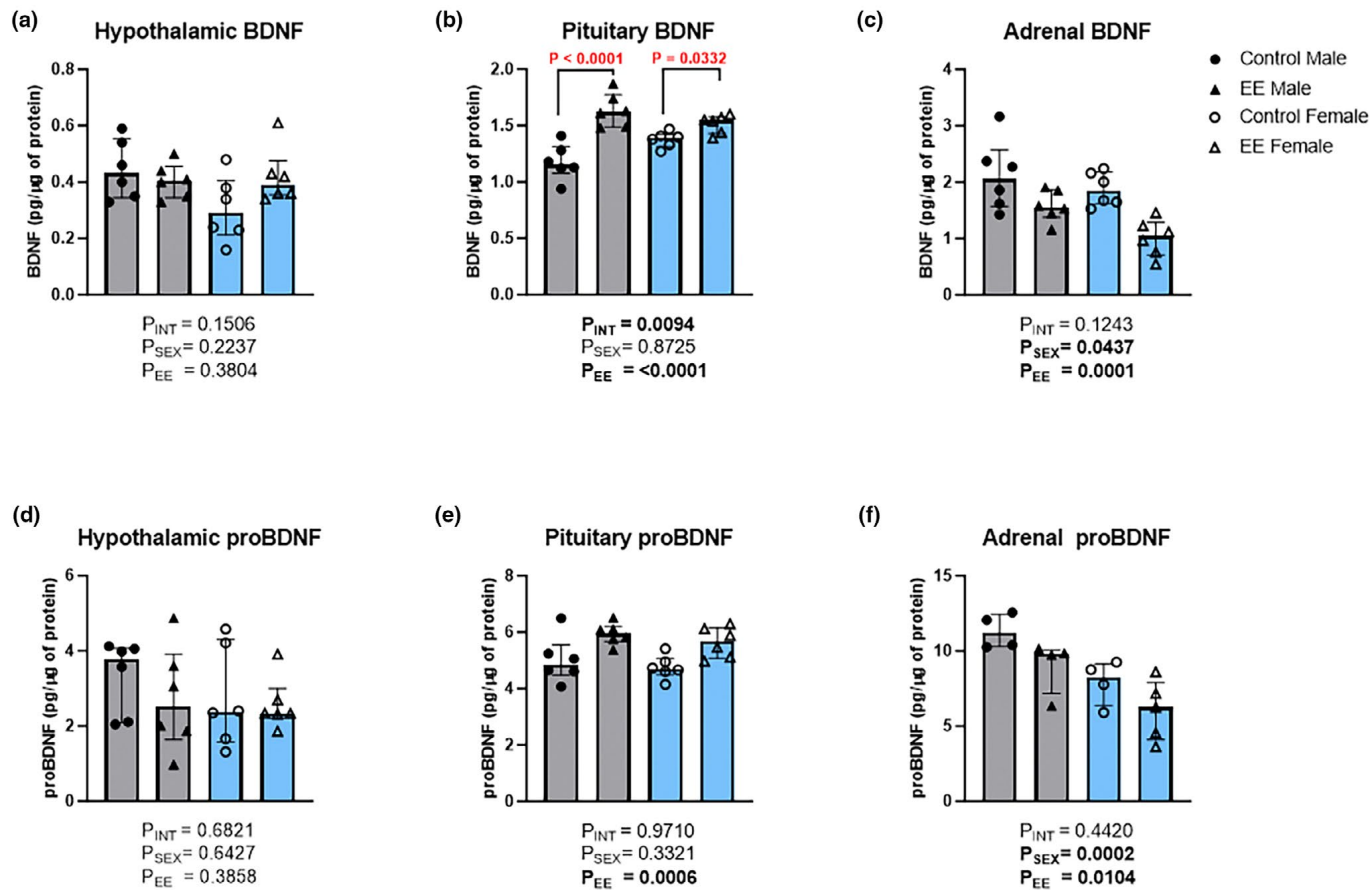
The effects of environmental enrichment (EE) on the expression levels of BDNF and proBDNF in the hypothalamus, pituitary and adrenal glands of male and female mice. Data for hypothalamic BDNF (a), pituitary BDNF (b), adrenal BDNF (c), hypothalamic proBDNF (d), pituitary proBDNF (e), and adrenal proBDNF (f) are shown. Sheirer‐Ray‐Hare test with Sidak's multiple comparisons. Data are presented as median (IQR). Males are shown in gray and females in blue. Hypothalamic brain‐derived neurotrophic factor (BDNF) for both sexes: Control *n* = 6; EE *n* = 6. Hypothalamic proBDNF for both sexes: Control *n* = 6; EE *n* = 6. Pituitary BDNF for both sexes: Control *n* = 6; EE *n* = 6. Pituitary proBDNF for both sexes: Control *n* = 6; EE *n* = 6. Adrenal BDNF for both sexes: Control *n* = 6; EE *n* = 6. Male adrenal proBDNF: Control *n* = 4; EE *n* = 4. Female adrenal proBDNF: Control *n* = 4; EE *n* = 5. Small differences in sample sizes were due to inadequate sample volumes and technical issues with tissue collection.

BDNF and proBDNF levels were also measured in the hypothalamus, pituitary gland and adrenal gland. Analysis of hypothalamic BDNF levels showed no significant main effects of sex (Figure 6a; H(1) = 1.47, *p* = 0.2237), environment (H(1) = 0.75, *p* = 0.3804) or an interaction (H(1) = 2.09, *p* = 0.1506). A significant main effect of environment (Figure 6b; H(1) = 14.97, *p* < 0.0001), but not sex (H(1) = 0.01, *p* = 0.8725), was evident for pituitary BDNF, as well as a significant interaction effect (H(1) = 2.34, *p* = 0.0094). Post‐hoc analysis showed that male EE mice had higher pituitary BDNF levels (median = 1.62; IQR = 1.77–1.49; *p* < 0.0001) than their control counterparts (median = 1.16; IQR = 1.31–1.08). Similarly, female EE mice had higher pituitary BDNF levels (median = 1.55; IQR = 1.58–1.43; *p* = 0.0332) than female controls (median = 1.39; IQR = 1.43–1.34). Adrenal BDNF levels showed a significant main effect of sex (Figure 6c; H(1) = 2.17, *p* = 0.0437) and environment (H(1) = 10.27, *p* = 0.0001), but no significant interaction effect (H(1) = 1.20, *p* = 0.1243).

Analysis of proBDNF showed no significant main effects or interaction for the hypothalamus (Figure 6d; Sex: H(1) = 0.24, *p* = 0.6427; Environment: 0.85, *p* = 0.3858; Interaction: 0.19, *p* = 0.6821) and a significant main effect of treatment for the pituitary (Figure 6e; H(1) = 10.09, *p* = 0.0006), but not sex (H(1) = 0.61, *p* = 0.3321) and no significant interaction (H(1) = 0.00, *p* = 0.9710). In the adrenal gland, for proBDNF, there was a significant main effect of sex (Figure 6f; H(1) = 8.65, *p* = 0.0002) and environment (H(1) = 2.91, *p* = 0.0104); however, no significant interaction effect was found (H(1) = 0.21, *p* = 0.4420).

### 3.4 Catecholamine synthetic enzymes in the adrenal glands

**FIGURE 7:**
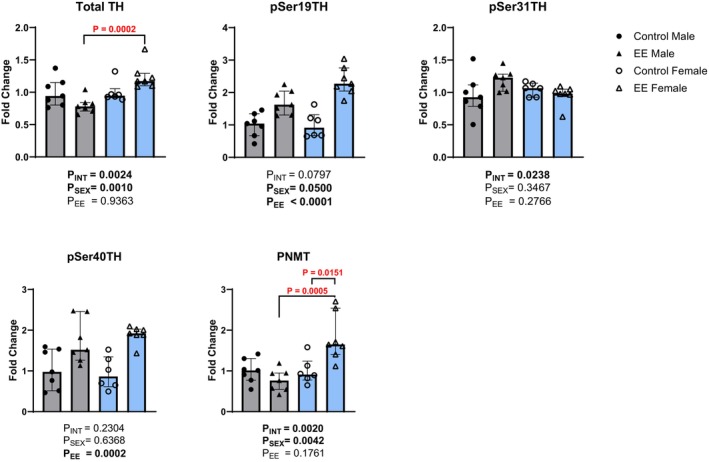
The effects of environmental enrichment (EE) on the regulation of catecholamine synthesis in the adrenal glands in male and female mice. Data for total TH (a), pSer19TH (b), pSer31TH (c), pSer40TH (d), and PNMT (e) are shown. Sheirer‐Ray‐Hare test with Sidak's multiple comparisons. Data are presented as median (IQR). Males are shown in gray and females in blue. For all analytes: Male control *n* = 7; Male EE *n* = 7; Female control *n* = 6; Female EE *n* = 7. Small differences in sample sizes were due to inadequate sample volumes and technical issues with tissue collection. TH, tyrosine hydroxylase; pSer19TH, pSer31TH, pSer40TH: Phosphorylation residues 19, 31 and 40 of tyrosine hydroxylase; PNMT, phenylethanolamine N‐methyltransferase.

Key proteins involved in the regulation of catecholamine biosynthesis were measured in the adrenal glands. Analysis of total TH expression showed a significant main effect of sex (Figure 7a; H(1) = 9.00, *p* = 0.0010), but not environment (H(1) = 0.00, *p* = 0.9363), with a significant interaction effect (H(1) = 7.38, *p* = 0.0024). Post‐hoc testing showed that female mice exposed to EE had higher total TH protein levels (median = 1.172; IQR = 1.29–1.10; *p* = 0.0002; *r = 0.999*) compared to males under the same conditions (median = 0.78; IQR = 0.84–0.71). To assess TH phosphorylation dynamics, levels of pSer19, pSer31, and pSer40 were also measured. Analysis of phosphorylation levels at pSer19TH showed a significant main effect of sex (Figure 7b; H(1) = 1.62, *p* = 0.0500) and environment (H(1) = 19.37, *p* < 0.0001), although the interaction effect did not reach significance (H(1) = 1.26, *p* = 0.0797). For pSer31 levels, there was a significant interaction effect (Figure 7c; H(1) = 5.86, *p* = 0.0238), although the effects of sex (H(1) = 0.92, *p* = 0.3467) and environment (H(1) = 1.24, *p* = 0.2766) did not reach significance. Post‐hoc comparisons showed no significant differences between groups. For pSer40TH, analysis showed a significant main effect of environment (Figure 7d; H(1) = 13.85, *p* = 0.0002), but not of sex (H(1) = 0.16, *p* = 0.6368) nor an interaction effect (H(1) = 1.05, *p* = 0.2304). Post‐hoc comparisons showed female EE mice had increased pSer40TH levels (median = 1.921; IQR = 2.10–1.88; *p* = 0.038) compared to female controls (median = 0.86; IQR = 1.35–0.61). Finally, analysis of adrenal PNMT levels showed a significant main effect of sex (Figure 7e; H(1) = 6.61, *p* = 0.0042), but not environment (H(1) = 1.28, *p* = 0.1761), with a significant interaction effect (H(1) = 8.00, *p* = 0.0020). Post‐hoc tests showed a significant increase in PNMT levels in female EE mice (median = 1.65; IQR = 2.54–1.40; *p* = 0.0151) compared to female controls (median = 0.91; IQR = 1.24–0.78). Female EE mice also had significantly higher PNMT levels (*p* = 0.0005) compared to male EE mice (median = 0.76; IQR = 0.95–0.55).

## 4 DISCUSSION

The present study investigated the effects of environmental enrichment (EE) on anxiety‐like behavior, neurotrophic factor expression and the catecholamine synthetic capacity of the adrenal glands in both male and female mice. This study highlights potential sex‐dependent effects of EE on behavioral outcomes and molecular outcomes. It was shown that female mice exposed to EE exhibited increased anxiety‐like behaviors in the OFT with increased adrenal catecholamine synthetic enzymes. Conversely, EE in male mice did not induce anxiety‐like behavior, with increased hippocampal and pituitary BDNF expression. Together, the present study suggests potential sex‐dependent modulation of anxiety‐like behaviors in EE mice with potential region‐specific modulation of molecular mechanisms related to these behaviors.

In particular, the behavioral data show that, in contrast to the widely held view of environmental enrichment as a beneficial or anxiolytic intervention, EE increased anxiety‐like behavior in the OFT in female mice as evidenced by a reduction in time spent in the central zone. According to Seibenhener and Wooten (2015), a preference of the peripheral zone in the OFT (and closed arms of the EPM) by mice is indicative of anxiety‐like behavior due to the natural tendency of mice to avoid large open areas that may bring danger. While many studies report anxiolytic effects of EE (Hüttenrauch et al., 2016; Leger et al., 2015; Meng et al., 2015; Ratuski et al., 2024; Resasco et al., 2021; Rojas‐Carvajal et al., 2020; Sakhaie et al., 2020), some studies have reported anxiogenic effects of EE (de Brito et al., 2025; dos Anjos‐Garcia et al., 2022). These inconsistencies in reported findings across different studies may be related to the use of different protocols of EE. The protocol used in the present study involved weekly rearrangement of EE items to prevent habituation; however, this may not have provided enough complexity to enrich the environment, since other studies rearranged enrichment items twice weekly or more (Leger et al., 2015; Meng et al., 2015; Rojas‐Carvajal et al., 2020). Recent reviews have also highlighted the need for standardization of EE protocols since different studies introduce different levels of novelty and complexity of environments in their protocols (Khalil, 2024; Lopes et al., 2017). A recent study which did not rearrange enrichment items in their protocol showed increased anxiety‐like behavior in both male and female mice (Dickson & Mittleman, 2021). Another interpretation could be that for the young post‐pubertal female mice in our study, once‐weekly rearrangement of enrichment items may have served as a chronic mild stressor, resulting in upregulation of molecular markers related to the stress response (discussed later). According to Sisk and Gee (2022) this period of adolescence to young adult mice is a period of high vulnerability where the HPA and sympathoadrenal medullary systems are highly sensitive to environmental conditions. Thus, what is perceived as ‘enrichment’ in one sex may be perceived as ‘unpredictable mild stress’ in the other, a hypothesis supported by the female‐specific adrenal adaptations observed in this study (discussed later).

Male mice, on the contrary, had more of an exploratory response following EE at least in the EPM since they showed a lower number of transitions between the closed arms and other zones in the test with no effect on locomotor measures, indicative of an exploratory phenotype. This reduction in transition frequency suggests a decrease in ’frantic’ exploratory reactivity often seen in standard‐housed mice which could suggest improved cognitive habituation to novel environments. These contrasting behavioral findings suggest sex‐dependent effects of EE on anxiety‐related behavior, at least with once‐weekly rearrangement of enrichment items possibly driven by different neurobiological pathways in each sex.

Indeed, the contrasting behavioral phenotypes observed were reflected in sex‐dependent molecular adaptations. In male mice, EE increased the expression of BDNF in the hippocampus. Since BDNF is known to promote neuroplasticity and emotional resilience (Buenrostro‐Jáuregui et al., 2025; Goseph et al., 2024), this upregulation may have contributed to the lack of anxiety‐like behavior, suggestive of a neuroprotective effect of EE. Meng et al. (2015) showed a similar increase in hippocampal BDNF levels in their study, with rearrangement of enrichment items every second day leading to anxiolytic effects on behavioral measures in male mice of a similar age. Thus, hippocampal BDNF regulation during EE may be influenced in a sex‐dependent manner.

BDNF and proBDNF levels were also analyzed in the hypothalamus, pituitary gland and adrenal gland. Here, EE resulted in the elevation of BDNF levels in the pituitary of male and female mice, which may reflect enhanced neurotrophic support in this key endocrine organ (Kumar et al., 2019). These results likely indicate enhanced expression of the BDNF gene due to the concomitant increased trend of proBDNF with BDNF possibly acting as a neurotrophic modulator of the HPA axis. Similar to our previous findings with exogenous corticosterone (Lin et al., 2022), increased pituitary neurotrophin expression may result in a compensatory or adaptive response to sustained glucocorticoid signaling or provide modulation to the hypothalamic–pituitary–thyroid axis or the hypothalamic–pituitary–gonadal axis (Mohamed et al., 2025; Przybył et al., 2021).

In female mice, there was a lack of a hippocampal BDNF response to EE, with female mice exposed to EE having lower levels than their male counterparts, as well as potential changes to peripheral stress response mechanisms. Specifically, BDNF levels in the adrenal glands showed significant main effects of both sex and environment, with a decreased trend in female mice. These results could suggest a loss of adrenal inhibitory regulation in females. While the role of adrenal BDNF is still being elucidated, the possible decrease here occurred alongside an upregulation of the catecholamine biosynthetic response of the adrenal glands. Our previous study showed that exogenous corticosterone had no effect on adrenal BDNF levels (Lin et al., 2022), suggesting that neurotrophic support of this peripheral organ may mediate functions other than glucocorticoid output such as adrenal catecholamine biosynthesis.

The demonstrated thigmotactic behavior by female mice in the OFT in our study was reflected by changes to adrenal enzymes involved in catecholamine biosynthesis. While studies that evaluated adrenal TH activity in female mice are lacking, we showed that the phosphorylation of TH was upregulated at the key phosphorylation sites pSer19TH and pSer40TH in female C57BL6J mice. Furthermore, an increase was seen in PNMT levels in female mice exposed to EE, suggesting enhanced biosynthetic capacity for both noradrenaline and adrenaline, which may have also contributed to a heightened stress‐driven behavioral response in these mice, although future studies should confirm this with the concurrent measurement of adrenaline or noradrenaline. In male mice, EE had no effects on catecholamine‐related proteins. This is in contrast with a previous study by Marashi et al. (2003) that showed EE increased adrenal TH activation in male mice, which may indicate that our protocol conferred resilience to stress in males. The inconsistencies in these findings may be due to differences in the strain of mice since Marashi et al. (2003) used an inbred strain of CS mice, although they were of similar age. Together, these findings may suggest that female mice possess a greater adrenomedullary response to EE.

This female‐specific effect on adrenal catecholamine biosynthetic enzymes may be attributed to the modulatory effects of female sex hormones on the sympathoadrenal medullary system. Estrogen is known to enhance the transcription and phosphorylation of TH via estrogen receptors present in the adrenal medulla (Khasar et al., 2005; Yanagihara et al., 2006). This hormonal influence likely lowers the threshold for adrenal medullary activation, rendering the female sympathoadrenal medullary system more reactive to environmental changes which could have contributed to female mice perceiving the novelty brought about by our EE protocol as a mild chronic stressor rather than a cognitive benefit. This was further supported by the lack of hippocampal BDNF upregulation in females.

Thus, EE may serve as a mild stressor in female C57Bl6J mice since the findings of this study suggest female mice had increased anxiety‐like behavior, while adrenal enzymes involved catecholamine synthesis were increased. Conversely, EE in male mice may have enhanced neurotrophic support in the hippocampus with minimal impacts on adrenal catecholamine synthetic enzymes. This study highlights the necessity of measuring peripheral enzymatic markers alongside central growth factors to better capture the impact of housing on animal welfare and shows that EE is a highly nuanced intervention that cannot be universally applied across different sexes and species.

This study had several limitations which should be noted. Firstly, the sample sizes used across the behavioral and molecular analyses were small, thus non‐parametric statistical analyses were carried out due to heterogeneity in the data, limiting statistical power. Another limitation may have been that the EE protocol used in the present study may differ in complexity and duration in comparison to models used in other studies, making comparisons across studies difficult, although it should be noted that this is a current issue with EE models (Khalil, 2024; Ratuski & Weary, 2022). Behavioral testing was carried out during the light phase during which rodents are less active; therefore, this may have confounded the reported behavioral outcomes since locomotor activity and exploration may have been reduced. The present study also did not measure circulating levels of corticosterone nor catecholamines such as adrenaline; therefore, the functional significance of alterations in the adrenal biosynthetic enzymes remains uncertain. Thus, it is not clear whether or not alterations to neurotrophic support in the HPA axis and catecholamine synthetic enzymes in the adrenal glands ultimately led to activation of the major stress response systems involved. Future studies should investigate downstream receptor signaling, such as TrkB and p75NTR pathways to better elucidate the alterations in BDNF and proBDNF levels in response to EE. The measurement of HPA axis or adrenomedullary outputs such as corticosterone and adrenaline may strengthen interpretations in this context. Finally, a further limitation of this study was that the oestrous stage was not monitored in female mice during the experiments and behavioral testing; thus, it is not clear whether this had any influence over the sex differences identified in this study. However, random allocation of animals to experimental groups likely ensured distributed representation of cycle stages across both control and EE conditions, mitigating the risk that the observed anxiogenic phenotype was driven by cycle synchronization within a group. Furthermore, the high degree of consistency between the behavioral findings and upregulation of adrenal biosynthetic enzymes is suggestive of a robust environmental effect rather than a transient effect of the oestrus cycle. Future studies evaluating sex differences in EE should consider the potential effects of the stage of the oestrous cycle on behavioral outcomes as this could potentially aid in the identification of a ‘window of vulnerability’ in female mice exposed to EE given the conflicting findings in the limited studies assessing female mice.

## 5 CONCLUSION

Our findings suggest that EE induced sex‐dependent responses in mood‐related behavior as well as in neurotrophin expression in the brains of C57BL6J mice. EE increased anxiety‐like behaviors in females, along with increased levels of catecholamine synthetic enzymes in the adrenal glands. Taken together, these results suggest that EE may have served as a mild stressor in female mice. Conversely, EE increased hippocampal BDNF in male mice without alterations to adrenal catecholamine synthetic enzymes, suggesting that male mice may be more stress‐resilient in a stimulating environment. Thus, these findings highlight the nuanced nature of EE as an intervention and the importance of considering sex as a biological variable in translational neuroscientific research. Future studies investigating how EE differentially modulates catecholamine synthesis may provide valuable insight into sex‐specific mechanisms of vulnerability or resilience relevant to anxiety disorders.

## AUTHOR CONTRIBUTIONS

**M. F. Herselman:** Formal analysis; software; validation; visualization. **S. Y. Luo:** Data curation; investigation. **L. Y. Lin:** Data curation; investigation; methodology; project administration; software; validation. **X. F. Zhou:** Resources. **L. Bobrovskaya:** Conceptualization; investigation; methodology; resources; supervision.

## FUNDING INFORMATION

This work to Liying Lin: This work did not receive any specific grant from agencies in the public, commercial, or not‐for‐profit sectors. All authors declare no conflict of interest.

## CONFLICT OF INTEREST STATEMENT

None.

## Data Availability

The data that support the findings of this study are available from the corresponding author upon reasonable request.
